# Utilization and outcomes of transcranial magnetic stimulation and usual care for MDD in a large group psychiatric practice

**DOI:** 10.1186/s12888-024-05928-4

**Published:** 2024-07-09

**Authors:** Jesse Bastiaens, Natalie Brown, Richard A. Bermudes, Jessie L. Juusola, Dena M. Bravata, Tobias F. Marton

**Affiliations:** 1 Mindful Health Solutions, 360 Post St #500, San Francisco, CA 94108 USA; 2 Department of Psychiatry, University of California, San Francisco, CA USA; 3 Anchor Outcomes, San Francisco, CA USA; 4 Center for Primary Care & Outcomes Research, Stanford University, Palo Alto, CA USA

**Keywords:** MDD, Transcranial magnetic stimulation, Treatment-resistant depression

## Abstract

**Background:**

General psychiatrists’ practice standards vary regarding when to implement transcranial magnetic stimulation (TMS) for care of patients with major depressive disorder (MDD). Furthermore, few studies have examined real-world utilization and clinical outcomes of TMS. This study analyzed data from a large, multi-site psychiatric practice to evaluate utilization and outcomes of TMS as well as usual care (UC) for patients with MDD.

**Methods:**

Depression outcomes for TMS and UC among adult patients at a multi-site psychiatric group practice were examined in this retrospective cohort analysis. Patients with a primary diagnosis of MDD, PHQ-9 ≥ 10, and a visit in November 2020 with 6-month follow-up were included and categorized into the TMS or UC cohorts.

**Results:**

Of 1,011 patients with qualifying PHQ-9 at the baseline visit, 9% (89) received a full course of TMS, and 583 patients receiving UC met study inclusion criteria (339 patients were excluded due to lacking a 6-month follow-up visit or receiving esketamine during the study period). The TMS cohort had higher baseline PHQ-9 than UC (17.9 vs. 15.5, *p* < .001) and had failed more medication trials (≥ 4 vs. 3.1, *p* < .001). Mean PHQ-9 decreased by 5.7 points (SD = 6.7, *p* < .001) in the TMS cohort and by 4.2 points (SD = 6.4, *p* < .001) in the UC cohort over the study period. Among patients who had failed four or more antidepressant medications, PHQ-9 decreased by 5.8 points in the TMS cohort (SD = 6.7, *p* < .001) and by 3.2 points in the UC cohort (SD = 6.3, *p* < .001).

**Conclusions:**

TMS utilization was low, despite TMS showing significant real-world clinical benefits. Future research should examine and address barriers to wider adoption of TMS into routine patient care for patients with treatment-resistant MDD. Wider adoption including routine use of TMS in less treatment-resistant patients will allow statistical comparisons of outcomes between TMS and UC populations that are difficult to do when TMS is underutilized.

## Background

Mental illness is increasingly prevalent, currently affecting nearly one in five adults in the United States [1]. The health and economic impact of mental illness is substantial; depression in particular is the single largest contributor to disability worldwide [2]. In the United States, the economic burden of major depressive disorder (MDD) was estimated to be $326.2 billion in 2018, accounting for direct costs, suicide-related costs, and workplace costs [3]. While many antidepressant therapies are available, fewer than 50% of patients respond to first line medication, and chances of response and remission decrease with subsequent medication trials [4].

Transcranial magnetic stimulation (TMS) has been demonstrated to be an effective therapy for several mental health disorders [5–8]. It was first provided clearance by the United States Food and Drug Administration (FDA) for treating patients with MDD who failed to improve after one antidepressant and has been demonstrated to be effective in treatment-resistant populations [7, 8]. It has since been cleared by the FDA for treating obsessive compulsive disorder (OCD) and depression with anxious features, and the evidence base demonstrating its efficacy continues to grow [6, 9].

In 2017, consensus recommendations for clinical use of TMS for MDD were published [10]. However, despite a growing evidence base indicating that TMS should be introduced early in the care of patients with MDD, general psychiatrists in community practice have not yet adopted a consistent standard for when TMS should be introduced into the MDD treatment pathway, and it remains underutilized [11]. Several factors likely contribute to this inconsistent and low utilization, including limited TMS curriculum in psychiatry training programs, limited access to TMS devices, and inconsistent and restrictive payer coverage of TMS therapy [11–13]. Additionally, there are a number of siloed TMS-focused specialty clinics, which may create additional barriers for patients, the majority of whom are treated in general psychiatric practices that only offer medication management and psychotherapy [14]. Low utilization in community practice has resulted in a dearth of studies examining clinical outcomes of TMS in real world settings, or comparing to outcomes with usual care (UC), which in turn has further exacerbated the cycle of limited utilization.

Given the demonstrated efficacy of TMS and the shortcomings of standard antidepressant therapies, it is important to facilitate earlier and wider use of TMS for patients with MDD. As such, the purpose of this study was to leverage data from a multi-site psychiatric group practice that is unique in offering TMS alongside general psychiatric care (i.e., medication management and psychotherapy) in order to build the literature base on real-world TMS utilization rates and outcomes. We first quantified TMS utilization in this practice setting, and then we examined clinical outcomes for patients with MDD receiving TMS and for those receiving UC. We characterized clinical outcomes based on Patient Health Questionnaire (PHQ-9) scores collected by the practice before, during, and after treatment [15].

## Methods

### Study design

We conducted a retrospective cohort analysis of data collected during routine delivery of mental health services at a multi-site psychiatric group practice (Mindful Health Solutions, San Francisco, CA, USA). Treatment modalities utilized at the practice include psychotherapy, medication management, TMS, and intranasal esketamine. Data from adult patients with a primary diagnosis of MDD and a clinic or telehealth visit in November 2020, without record of receiving TMS in the month of October 2020, were considered for inclusion. We excluded patients with a baseline PHQ-9 below 10, who lacked a follow-up visit between April to June 2021 (6 months ± 1 month from their baseline visit), or who received esketamine during the study period. Demographic information, utilization metrics, and outcome measures were extracted from electronic medical records for all eligible patients. Since the study was a secondary analysis of previously collected, de-identified data, it was deemed exempt from ethics oversight by the WCG Institutional Review Board (Protocol No. MHS.001, February 10, 2023).

### Study cohorts

Patients were categorized into two mutually exclusive cohorts, UC and TMS. Usual care consisted of conventional pharmacotherapy with or without concurrent psychotherapy. Patients who received at least 25 TMS treatments between November 2020 and June 2021, in tandem with conventional pharmacotherapy, were assigned to the TMS cohort. Patients who received less than a full course of TMS during the study period (i.e., fewer than 25 treatments) were assigned to the UC cohort. The stimulation protocols used in TMS treatments varied, following community standards, using one of three commercially available TMS devices (BrainsWay Deep TMS, Burlington, MA, USA; Neuronetics NeuroStar Advanced Therapy, Malvern, PA, USA; MagVenture TMS Therapy, Alpharetta GA, USA). Across both cohorts, we subcategorized patients according to whether they were considered severely treatment-resistant, defined as having failed four or more trials of antidepressant pharmacotherapy during the current depressive episode [4, 16–18].

### Outcome measures

We measured utilization of TMS in the study sample as the percentage of MDD patients with PHQ-9 of 10 or higher at baseline who received a full course of TMS. We included patients who did not have a qualifying follow-up visit in the study period in this calculation to more accurately represent the presenting population for whom TMS could be considered.

Clinical outcomes for MDD patients were characterized based on the well-validated PHQ-9 measure of depression severity [15]. Our primary outcome of interest was the mean change in PHQ-9 score over the 6-month study period for each cohort, from the baseline visit in November 2020 to the follow-up visit (the visit between April to June 2021 that was closest to 6 months after the baseline visit). For the TMS cohort, we also calculated the change in PHQ-9 from first TMS treatment to the end of the TMS course, which was prior to the 6-month follow-up visit. We also calculated response rates, clinical improvement rates, and remission rates for each cohort. Response rate was defined as the percentage of the cohort with ≥ 50% improvement in PHQ-9 score from baseline to follow-up visit or from start to end of the TMS course. Clinical improvement rate was defined as the percentage of the cohort with a decrease of at least 5 points on the PHQ-9 from baseline to follow-up visit or from start to end of the TMS course [19]. Remission rate was calculated as the percentage of the sample with a PHQ-9 score below 5 at the follow-up visit or end of the TMS course [19].

### Statistical analysis

Descriptive statistics are presented to describe the demographics and baseline health status of participants. We compared baseline characteristics in the TMS cohort with the UC cohort using 2-tailed unpaired t-tests for continuous measures and chi square tests for categorical measures. For the outcomes of mean change in PHQ-9 score in each cohort over the study period or over TMS treatment period, we conducted 2-tailed paired t-tests. All analyses were conducted in Microsoft Excel Version 2304 Build 16.0, with an alpha of 0.05 for assessment of statistical significance.

## Results

### Sample characteristics

**Fig. 1:**
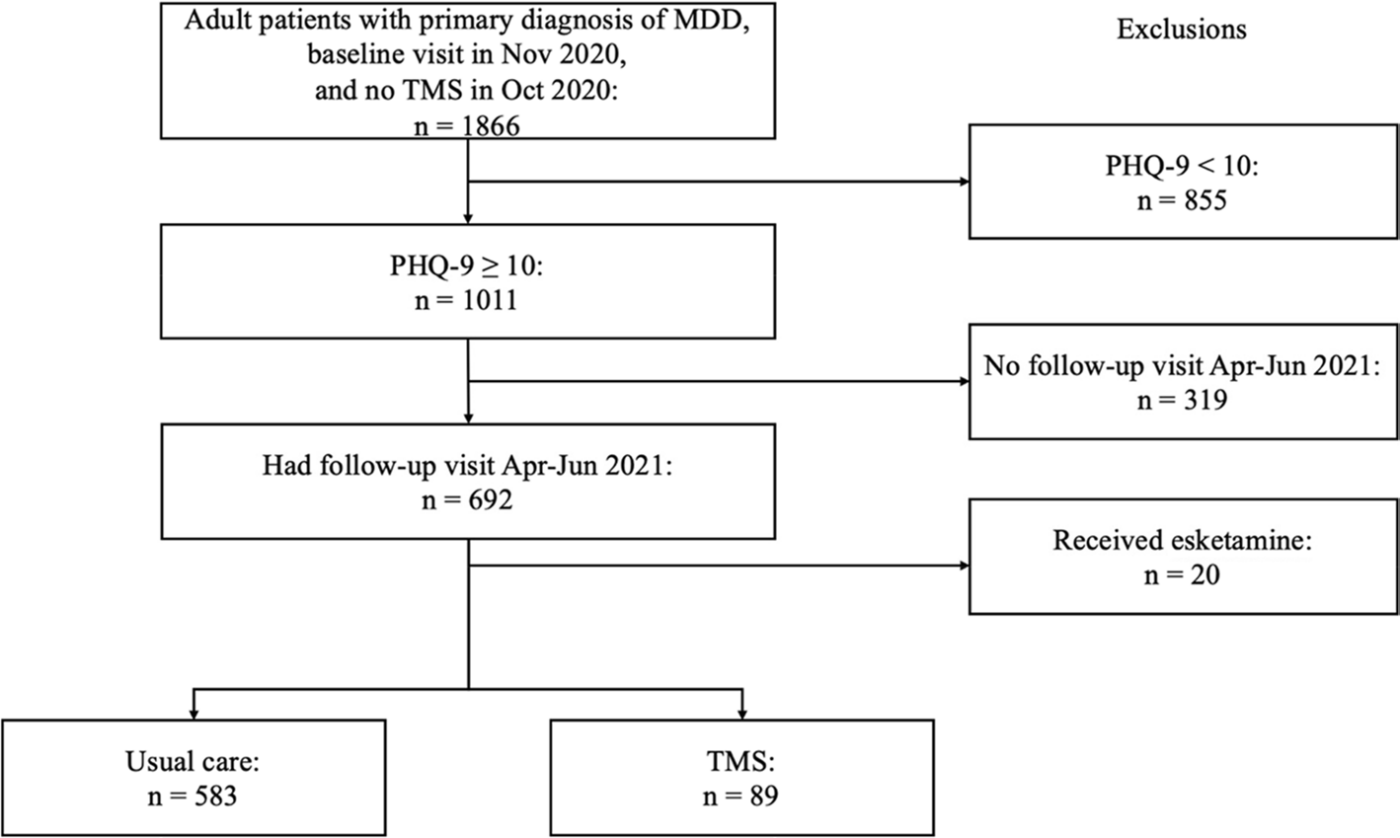
Study inclusion flow. Abbreviations: MDD = major depressive disorder, PHQ-9 = Patient Health Questionnaire, TMS = transcranial magnetic stimulation

Of the 1,866 adult patients with MDD who were considered for inclusion, 1,011 had baseline PHQ-9 of 10 or greater (Fig. 1). Of those patients, 89 (9%) received a full course of TMS during the study period. All patients in the TMS cohort also met the inclusion criteria of having a follow-up visit between April to June 2021, while only 583 patients in the UC cohort met the criteria. Nine patients in the UC cohort (1.5%) did receive some TMS (< 25 treatments) during the study period. Treatment was provided by 44 clinicians (38 MDs, 6 NPs) at 13 sites across California, with each clinician providing TMS having over 20 h of structured TMS training and supervision. The majority of the TMS delivered was by clinicians with greater than 4 years of experience using TMS to treat MDD.

The TMS and UC cohorts were similar across gender, insurance type, and secondary diagnoses (Table 1). Nearly two-thirds of patients were female, almost 90% had commercial insurance, and the most common secondary diagnosis was generalized anxiety disorder (GAD) (Table 1). The TMS cohort differed from the UC cohort in that patients were on average older (43.2 years vs 39.3 years, *p* = 0.02), had more severe depression symptoms at baseline (PHQ-9 of 17.9 vs. 15.5, *p* < 0.001), and had failed more medication trials (> 4 vs. 3.1, *p* < 0.001) (Table 1). Previous treatment history of TMS was similar across the cohorts (27% vs. 22%, *p* = 0.29) (Table 1).

**Table 1:** Sample characteristics by cohort and overall

Characteristic	UC (*n* = 583)	TMS (*n* = 89)	Total (*n* = 672)	*P* (UC vs TMS)
**Demographic**				
Age, mean (SD), y	39.3 (13.9)	43.2 (14.1)	39.9 (14.0)	.02
Sex, % (n)				.56
Female	65.0 (379)	60.7 (54)	64.4 (433)	
Male	30.5 (178)	32.6 (29)	30.8 (207)	
Not reported	4.5 (26)	6.7 (6)	4.8 (32)	
Insurance type, % (n)				.66
Commercial	89.7 (523)	85.4 (76)	89.1 (599)	
Medicare	4.8 (28)	6.7 (6)	5.1 (34)	
Cash pay	1.0 (6)	1.1 (1)	1.0 (7)	
Unknown	4.5 (26)	6.7 (6)	4.8 (32)	
**Clinical**				
Secondary diagnosis, % (n)				
GAD	47.0 (274)	41.5 (37)	46.3 (311)	.34
Anxiety NOS	8.1 (47)	5.6 (5)	7.7 (52)	.42
PTSD	5.0 (29)	3.4 (3)	4.8 (32)	.51
PHQ-9 score at baseline, mean (SD)	15.5 (4.5)	17.9 (4.7)	15.8 (4.6)	< .001
No. of failed medications at baseline, mean^a^	3.1	4.0^b^	3.2	< .001
Had previous course(s) of TMS, % (n)	22 (128)	27 (24)	23 (152)	.29

*Abbreviations: GAD* Generalized anxiety disorder, *NOS* Not otherwise specified, *PHQ-9* Patient Health Questionnaire, *PTSD* Post-traumatic stress disorder, *SD* Standard deviation, *TMS* Transcranial magnetic stimulation, *UC* Usual care

^a^Number of failed medications was only tracked as 0, 1, 2, 3, and 4 + , so a standard deviation cannot be accurately calculated

^b^Because number of failed medications was only tracked as 0, 1, 2, 3, and 4 + , the mean should be interpreted as 4 +

For the TMS cohort, the full course of TMS treatment was generally shorter than the 6-month study period. The first TMS treatment occurred on average 54 days (SD = 57) after the baseline visit in November 2020. The full treatment course averaged 37 treatments (SD = 8) and spanned 70 days (SD = 29), and the average time from the final TMS treatment to the follow-up visit was 102 days (SD = 56).

### Clinical outcomes

**Fig. 2:**
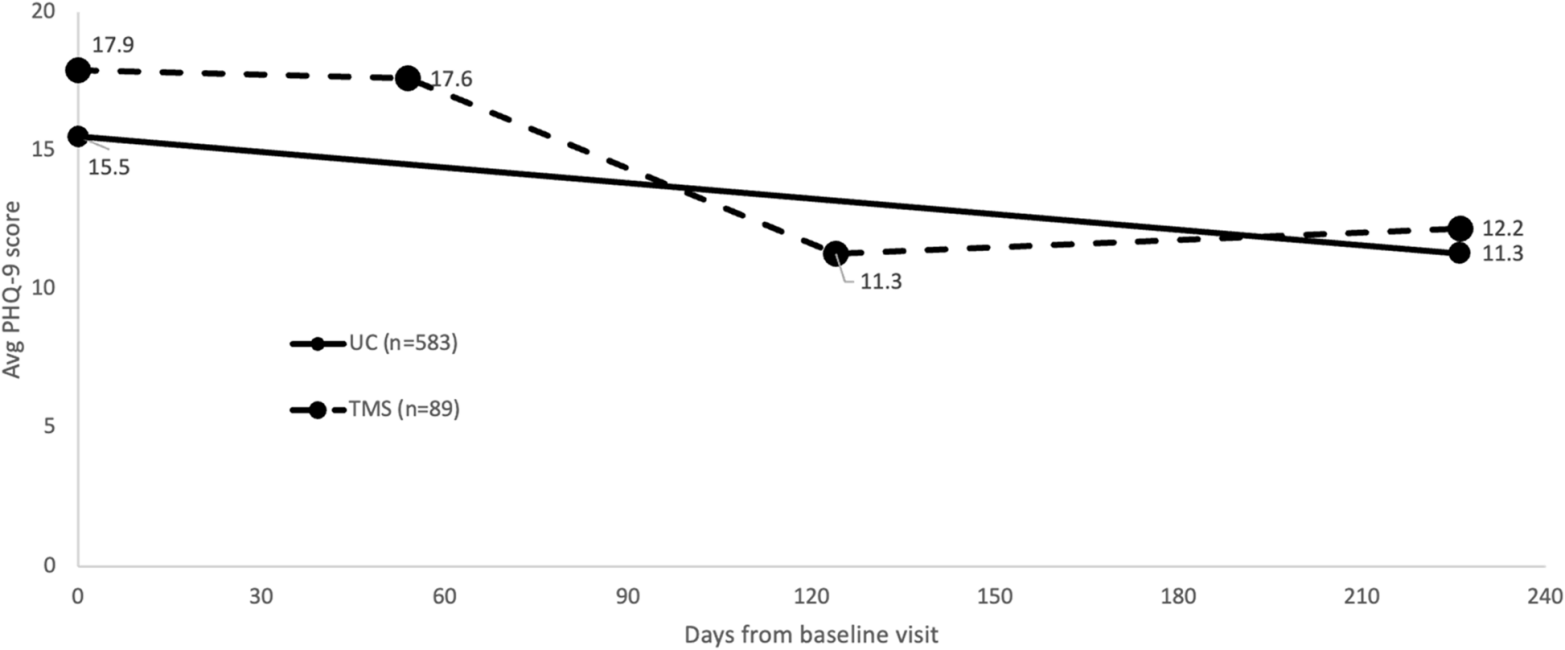
Change in PHQ-9 over study period by cohort. (TMS cohort had PHQ-9 data points at study baseline, start of TMS treatment which was on average 54 days after the baseline visit, end of TMS treatment which was on average 70 days after the start, and at the follow-up visit). Abbreviations: PHQ-9 = Patient Health Questionnaire, TMS = transcranial magnetic stimulation, UC = usual care

**Fig. 3:**
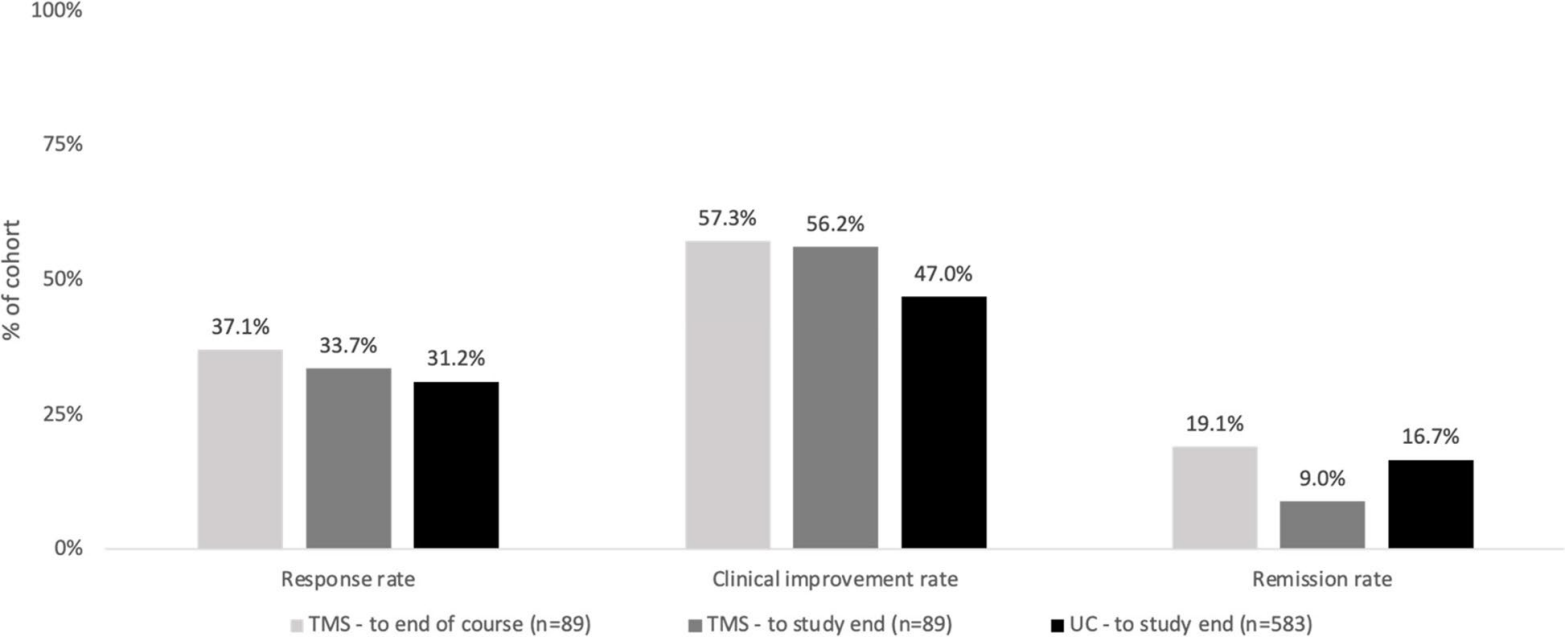
Clinical outcomes by cohort (Response rate is the percentage of the cohort with ≥ 50% improvement in PHQ-9 score from baseline to follow-up visit or from start to end of the TMS course. Clinical improvement rate is the percentage of the cohort with a decrease of ≥ 5 points on the PHQ-9 from baseline to follow-up visit or from start to end of the TMS course. Remission rate is the percentage of the sample with a PHQ-9 score < 5 at the follow-up visit or end of the TMS course). Abbreviations: TMS = transcranial magnetic stimulation, UC = usual care

From baseline to the end of the study period, average PHQ-9 decreased by 4.2 points (SD = 6.4, *p* < 0.001) in the UC cohort and by 5.7 points (SD = 6.7, *p* < 0.001) in the TMS cohort (Fig. 2, Table 2). Over the TMS treatment course, we found an average PHQ-9 decrease of 6.3 points from treatment start to end (SD = 6.2, *p* < 0.001) (Fig. 2, Table 2). Treatment response rates and clinically meaningful improvement rates were higher for the TMS cohort at the end of the TMS treatment course than at study end (37.1% vs 33.7% and 57.3% vs 56.2% respectively; Fig. 3, Table 3). Response and clinical improvement rates in the UC cohort were 31.2% and 47.0% respectively (Fig. 3, Table 3). In the TMS cohort, the remission rate was 19.1% at end of TMS treatment but decreased to 9.0% by the end of the study period, while in the UC cohort, the remission rate was 16.7% at study end (Fig. 3, Table 3).

**Table 2:** Clinical outcomes by cohort and for treatment-resistant subgroup. Mean PHQ-9 and change over time

	Total Sample	Treatment-Resistant Subgroup
**UC – PHQ-9, mean (SD)**	***n* = 583**	***n* = 258**
Baseline	15.5 (4.5)	16.2 (4.8)
Study end	11.3 (6.3)	13.0 (6.6)
Change from baseline to study end	4.2 (6.4) *p* < .001	3.2 (6.3) *p* < .001
**TMS – PHQ-9, mean (SD)**	***n* = 89**	***n* = 84**
Baseline	17.9 (4.7)	18.0 (4.8)
TMS start	17.6 (4.5)	17.7 (4.6)
TMS end	11.3 (6.3)	11.2 (6.4)
Study end	12.2 (5.9)	12.1 (5.8)
Change from TMS start to TMS end	6.3 (6.2) *p* < .001	6.5 (6.2) *p* < .001
Change from baseline to study end	5.7 (6.7) *p* < .001	5.8 (6.7) *p* < .001

*Abbreviations: PHQ-9* Patient Health Questionnaire, *SD* Standard deviation, *TMS* Transcranial magnetic stimulation, *UC* Usual care

**Table 3:** Clinical outcomes by cohort and for treatment-resistant subgroup. Response, clinical improvement, and remission rates^a^

	UC	TMS	
		To TMS end	To study end
**Total sample**	***n* = 583**	*n*** = 89**	
Response rate, %	31.2	37.1	33.7
Clinical improvement rate, %	47.0	57.3	56.2
Remission rate, %	16.7	19.1	9.0
**Treatment-Resistant Subgroup**	***n* = 258**	***n* = 84**	
Response rate, %	23.3	38.1	34.5
Clinical improvement rate, %	39.9	58.3	56.0
Remission rate, %	12.4	20.2	8.3

*Abbreviations: TMS* Transcranial magnetic stimulation, *UC* Usual care

^a^Response rate is the percentage of the cohort with ≥ 50% improvement in PHQ-9 score from baseline to follow-up visit or from start to end of the TMS course. Clinical improvement rate is the percentage of the cohort with a decrease of ≥ 5 points on the PHQ-9 from baseline to follow-up visit or from start to end of the TMS course. Remission rate is the percentage of the sample with a PHQ-9 score < 5 at the follow-up visit or end of the TMS course

When examining the subset of patients who were categorized as severely treatment-resistant (i.e., 4 or more antidepressant failures), improvements in the TMS cohort were similar to those in the overall cohort. For patients receiving UC, improvements were lower than in the overall cohort. The majority of the TMS cohort was severely treatment-resistant (94.4%) as compared to 44.3% of the UC cohort. In this subset of patients, average PHQ-9 decreased by 3.2 points in the UC cohort (SD = 6.3, *p* < 0.001) and by 5.8 points in the TMS cohort over the study period (SD = 6.7, *p* < 0.001) (Table 2). Over the TMS treatment course, average PHQ-9 decreased by 6.5 points (SD = 6.2, *p* < 0.001) (Table 2). Response, clinical improvement, and remission rates showed similar trends, being similar for the severely treatment-resistant subset of the TMS cohort and being lower for the severely treatment-resistant subset of the UC cohort. At study end, the response rate in the severely treatment-resistant TMS cohort was 34.5% versus 38.1% at the end of the TMS course, and clinical improvement rates were 56.0% at study end versus 58.3% at treatment end (Table 3). In the severely treatment-resistant subset of the UC cohort, response and clinical improvement rates were 23.3% and 39.9% respectively (Table 3). At the end of the TMS treatment course, 20.2% of the cohort were in remission, compared to 8.3% by study end, and the remission rate in treatment-resistant patients in the UC cohort was 12.4% at study end (Table 3).

## Discussion

In this real-world study of a large sample of patients with MDD, TMS patients experienced statistically significant improvements in depression severity as measured by the PHQ-9, and more than half of TMS patients saw a clinically meaningful response. Usual care patients also experienced statistically significant improvements in depression severity over the study period. Due to the differences in depression severity and treatment resistance at baseline between the cohorts, we were unable to statistically compare the levels of improvement between the cohorts. Additionally, TMS utilization was low in this setting, despite producing clinically meaningful outcomes. This underutilization led to attempts at analyzing sub-cohorts matched on baseline depression severity to be underpowered.

In the subpopulation that had failed four or more antidepressants, which represented nearly all the patients in the TMS cohort and 44.3% of the UC cohort, improvement was similar to the overall cohort for TMS patients but lower than the overall cohort for UC patients. This diminished efficacy of UC in patients who are treatment resistant as compared to those who are not treatment resistant aligns with outcomes reported in both the STAR*D trial and the broader TMS literature, which demonstrate that response rates to additional antidepressant trials drop significantly after two medication failures, whereas TMS efficacy is largely maintained in more resistant populations [4, 8].

This dataset is unique in that it is from a practice that offers many modalities of treatment in its approach to patient care. Whereas most mental health practices are either general psychiatry practices that do not offer TMS or specialized practices that only offer TMS and not general psychiatric care, this practice offers both UC and TMS. Even in this setting, less than 10% of potentially eligible patients received a full course of TMS during the study period. This is in large part due to insurance coverage limitations. At the time of the study analysis window, most health insurers required four failed medication trials before approving TMS, despite FDA clearance for use after one failed antidepressant trial as well as evidence that TMS is more efficacious and cost effective in patients with low levels of treatment resistance [20, 21]. This disconnect between evidence and insurance coverage policies persists, as examined in a recent commentary article, and leads to use being unnecessarily restricted to very sick populations [11].

Insurance coverage restrictions factor into the time to treatment for patients in multiple ways. First, the time required to trial additional medications may be reflected in the older age of TMS patients; in this study, patients in the TMS cohort were significantly older than those who received UC. From the patient standpoint, this is additional years of suffering, which contributes to the economic burden of MDD. Commencing TMS earlier, after one failed antidepressant trial per FDA clearance, could improve patient outcomes earlier and in turn reduce the economic burden and unnecessary morbidity associated with MDD. Similarly, in this study we saw an average of 54 days from the baseline visit to the first TMS treatment. Some of this may be due to the time required to obtain insurance pre-authorization before scheduling treatment. For the patient, this is an additional 2 months of suffering. Updated health insurance coverage policies could lessen this burden.

Remission rates seen in the study were highest at the end of the TMS treatment course, with 19.1% of the TMS cohort experiencing remission at treatment end, and then decreasing over the average of 3 months from final TMS treatment to the follow-up visit to 9.0%. This is consistent with existing literature, as previous studies have shown that 50% of TMS responders relapse within one year, with the bulk of relapses occurring approximately 3 months after discontinuing treatment [22, 23]. The value of the time in remission for patients and their families cannot be understated, and especially in treatment-resistant patients, full sustained remission is very difficult to achieve [24]. Further, there is strong evidence that past response to TMS strongly predicts future response; therefore it is anticipated that the TMS-remitting patients who relapsed during the study period would have a high likelihood of response or remission with an additional course of TMS [25, 26]. On balance, we found that the significant and sustained reduction in symptom severity that TMS was able to achieve in this largely treatment-resistant population was clinically meaningful and as such, likely meaningful to the patients’ functioning.

Lastly, patients in the TMS cohort received an average of 37 TMS treatments, which represent somewhat longer TMS courses than those described in the extant literature, ranging typically from 20–30 treatments [27, 28]. That being said, a growing body of evidence supports improved efficacy with extended treatment courses, and most payors routinely approve 36 TMS treatments and may be willing to cover treatment extensions if clinically indicated [29].

### Limitations

This study had several limitations. First, the fact that it is a real-world sample, while important for producing real-world evidence, leaves the possibility of bias in the comparison. There may have been confounding factors that determined which patients received TMS. For example, patient preferences factor into which treatment is pursued, and this could influence response to treatment. Similarly, a patient’s previous experience with TMS could influence a psychiatrist’s decision to treat. However, in this sample, both cohorts had a similar percentage of patients with prior TMS experience, which may suggest that previous experience with TMS had little influence on decision to treat, but we did not have data on whether previous TMS treatments were successful.

Second, the study is limited by the small size of the TMS cohort as well as the data available to categorize treatment resistance. The limited use of TMS in only a very sick population hindered our ability to compare effectiveness between TMS and UC, as a sample matched on disease severity was too small to provide statistical power. Broader use of TMS would allow for more real-world comparison studies. Additionally, the electronic medical record used for this study only records the number of prior antidepressant treatment failures up to 4, and then utilizes a “4 or more” categorization. If the data were not truncated, the mean baseline number of medication failures in the TMS cohort would likely be higher than reported. Moreover, the relatively small TMS cohort (*n* = 89), did not provide the statistical power to categorize treatment resistance more granularly, into categories such as 1–2 failed medications vs 3–4 for example; future studies should examine whether number of failed medications impacts TMS effectiveness.

## Conclusions

This analysis contributes to the growing body of evidence demonstrating that TMS is an effective intervention for treatment resistant MDD. Despite the evidence of its efficacy as a depression therapy, utilization remains low, access is challenging, and health insurance coverage is restrictive. Facilitating wider adoption and earlier initiation of TMS for care of patients with MDD will lead to improved outcomes, provide the ability to compare these outcomes against those in UC, and holds promise for lowering the economic burden of depression. Further research is needed to examine the barriers to incorporating TMS into care and provide strategies for overcoming the barriers.

## Data Availability

The datasets analyzed during the current study are not publicly available due to privacy restrictions, but they may be made available from the corresponding author on reasonable request.

## Abbreviations

FDA: United States Food and Drug Administration
MDD: Major depressive disorder
OCD: Obsessive compulsive disorder
PHQ-9: Patient Health Questionnaire
SD: Standard deviation
TMS: Transcranial magnetic stimulation
UC: Usual care
